# Intertrigo

**Published:** 1890-02

**Authors:** 


### Intertrigo.

Intertrigo, or chafed skin, is such a common affection that it is generally overlooked, except, perhaps, the application of any available salve. Yet it is often painful and may under certain circumstances result in eczema. Again, the application of unmedicated ointments may do considerable harm by increasing the superficial inflammation and becoming rancid. A simple and most efficacious salve is the following, which we quote from the *Journal de Med.*, Nov. 10, 1889.

R Acid, borici..........gr viij

Lanolini..........℥ jss

Vaselini..........ʒ ijss

M. et fiat. unguentum.

